# A Bibliometric Review of Research on International Students’ Mental Health: Science Mapping of the Literature from 1957 to 2020

**DOI:** 10.3390/ejihpe11030056

**Published:** 2021-07-21

**Authors:** Quoc-Thai Cao, Quan-Hoang Vuong, Hiep-Hung Pham, Dinh-Hai Luong, Manh-Toan Ho, Anh-Duc Hoang, Minh-Trang Do

**Affiliations:** 1 EdLab Asia Educational Research and Development, Hanoi 100000, Vietnam; thaicao@edlabasia.org (Q.-T.C.); duc@edlabasia.org (A.-D.H.); 2 Center for Interdisciplinary Social Research, Phenikaa University, Hanoi 100000, Vietnam; hoang.vuongquan@phenikaa-uni.edu.vn (Q.-H.V.); toan.homanh@phenikaa-uni.edu.vn (M.-T.H.); 3 Center for Research and Practice on Education, Phu Xuan University, Hue 52000, Vietnam; 4 Vietnam National Institute of Educational Sciences, Hanoi 100000, Vietnam; luongdinhhai@vnies.edu.vn; 5 Thanh Do University, Hanoi 100000, Vietnam; minhtrang@researchcoach.edu.vn

**Keywords:** review, mental health, international student, science mapping, bibliometric review, COVID-19

## Abstract

The mental health of university students is not only a predominant topic for psychology and education researchers but also a source of interest for policymakers and various stakeholders. Although there has been a lot of research on higher education students’ mental health, we have little understanding on the mental health of international students (MHIS). Despite their distinctive characteristics compared to native students, the mental health issues of international students only started receiving attention very recently. So far, the literature on this topic lacks a comprehensive overview of its sub-topic and trending issues. By using bibliometric analysis, this research aims to fulfil this gap and provide a review of the extant literature about MHIS. Specifically, this study aims to (i) draw the growth trajectory and geographic distribution of the literature on MHIS; (ii) identify the documents and authors that have the greatest impact, generally and locally, within the major topic clusters of the literature on MHIS; and (iii) identify the most researched keywords in the literature on MHIS over time. The results have shown that academic documents about international students’ mental health are increasing in number and becoming more extensive content-wise. The research’s findings provide implications for stakeholders and identifies some prominent research avenues for the future.

## 1. Introduction

The mental health of university students is not only a predominant topic for psychology and education researchers but also a source of interest for policy-makers and various stakeholders [1]. Although there has been a lot of research on higher education students’ mental health, only a small portion of the literature was devoted to examining the mental health of international students (MHIS). Despite their distinctive characteristics compared to native students, the mental health issues of international students only started receiving attention very recently. So far, the literature on this topic lacks a comprehensive overview of its sub-topic and trending issues. As the trend of globalization increased in the education sector, the literature on the mental health of higher education students further expanded, which has brought the previously neglected group of international students into stronger focus [2]. As international students can contribute a large portion to the economy and later become efficient and qualified members of the workforce [3], innovations developed to ensure their mental wellness through promoting diversity and acculturation has become more explicitly prevalent in the educational policies of many countries and institutions [4].

However, researchers have found that previous results about local students’ mental health do not translate into the international sample, and a forced generalization might lead to poor policy-making decisions about international students’ mental health [5]. Due to the diverse cultural and social backgrounds that distinguish them from local students, research that provides the perspective of international students from different origins is very important to improve educational policy toward diversity and inclusion. According to Mori [6], international students not only suffered from common mental health issues among higher education students, such as depression, anxiety, loneliness, and stress, but also faced issues stemming from language barriers, acculturation, xenophobia, and racism, which can lead to serious consequences (e.g., suicide) if not addressed properly. Furthermore, since the COVID-19 outbreak (December 2019), international students are ever-more burdened with challenges such as low international support, loss of part-time jobs [7], heightened financial burden, unsupportive educational policies [8], and discrimination [9], which spark a stronger call for research on the mental wellbeing of international students. As the amount of research on international students’ mental health across different disciplines has been growing in recent years, new researchers on this topic might need a systematic outline of the literature as well as important related keywords about the topic. Therefore, this research acts as a blueprint that maps the literature about international students’ mental health in a well-established manner, which will hopefully reduce some of the difficulties hindering new researchers from dwelling on this topic as its emergency is heightening. The main research questions (RQ) that this research aims to answer are:

RQ1: What is the growth trajectory and geographic distribution of the literature on the MHIS?

RQ2: What documents and authors have had the greatest impact, generally and locally, within the major topic clusters of the literature on MHIS?

RQ3: What are the most researched keywords in the literature on MHIS over time?

In order to answer the above questions, an exploration of the Scopus databases was conducted by the authors. A descriptive analysis of the collected data was conducted with Excel and R (R studio, 2020), while the bibliometric analysis was conducted with VOSviewer.

## 2. Methods

In order to provide a reliable representation of the literature for future researchers, the papers examined were selected from Scopus-indexed journals. Scopus is one of the largest databases providing reliable and timely update indexing for journal publication, which suits the purpose of this research [10]. In general, the examination followed the “Preferred Reporting Items for Systematic Reviews and Meta-analysis”, which provided a systematic guideline for conducting meta-analysis and science mapping research [11,12,13]. The process can be summarized by four main steps

The first step involved identifying major keywords related to the topic of international students’ mental health. In order to perform the analysis, we first identified two groups of keywords that are relevant to international students and mental health, respectively. First, different synonyms of the keyword “international student” were listed, and we ended up with 17 keywords often used to refer to international students. Next, we added the relevant keywords that related to mental health problems. Here, we followed the practice conducted in the research of Storrie et al. [14], which identified six major keywords: social problems, emotional problems, psychiatric problems, mental problems, and medical or health. Lastly, we entered the keywords in the advanced searching section of Scopus. Overall, the keywords that have the same meaning, either referring to international students or mental problems, were separated by the argument “OR”, while the two keyword groups were connected with the argument “AND”, which resulted in the following search phrase:

TITLE-ABS-KEY ((“international student*” OR “overseas student*” OR “mobile student*” OR “mobilizing student*” OR “offshore student*” OR “outbound student*” OR “inbound student*” OR “exchange student*” OR “student exchange” OR “cross border student*” OR “student mobility” OR “international pedagog*” OR “study* abroad” OR “international exchange program*” OR “cross border education”) AND (“social*” OR “emotion*” OR “psych*” OR “mental”) AND (medical OR health OR problem))

As the goal in this stage was to find as many papers as possible, the only search exclusion imposed at this stage was for documents that were not in “English language”. The process yielded 957 documents that have been published from 1957 to date (15 October 2020).

**Figure 1:**
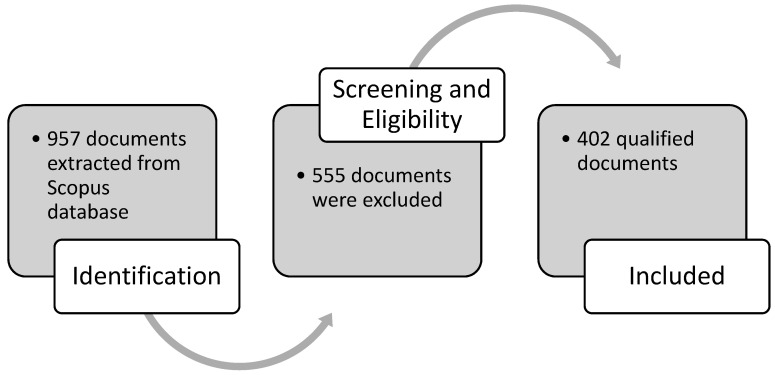
PRISMA diagram describing the data collection process of documents on MHIS from the Scopus database.

Thereafter, in the second step, the screening of document types was conducted. In this step, the examination of the titles and abstracts of the collected papers determined the degree of relevance to the topic of international students’ mental health. We included documents that discuss both the international student population and issues regarding their mental health when studying abroad. In this step, to ensure objectivity, two authors were assigned to conduct the examination process independently, compare their excluded papers, and came to a conclusion together. One author excluded 540 documents, while another excluded 552 documents. After a discussion on the exclusion criteria, a total number of 555 papers that were not explicitly related to international students’ mental health were excluded. The process led to a database of 402 qualified papers for analysis (see Figure 1).

In order to answer the research questions, a bibliometric analysis was adopted. Initiated by [15] in 1969, bibliometric analysis has become a powerful tool to analyze and synthesize the general trajectory and pattern of the researched topic in terms of bibliometric indicators such as the number of publications, the author’s country and affiliation, the number of citations per publication, the publication’s keywords, and so forth [16]. Within international student studies, in particular, some notable works using bibliometric analysis include [17,18].

Specifically, in order to answer the first and second research questions, the growth rate of the number of papers and the frequency of the most common keywords related to the topic were also calculated. While the descriptive analysis of the quantity of publications and keywords provided a general overview of the literature, citation analysis was being used to identify influential authors and papers within the domain of research [12]. The technique quantifies the frequency of the citation of authors and documents by authors and documents in the same domain of research, which subsequently answers the second research question. Regarding research question 3, co-citation analysis was used to uncover the intellectual structure of the research [19] on international students’ mental health and the method capable of revealing the authors that are frequently co-cited. Uncovering these different intellectual clusters might provide prospective researchers with an overview of major research paradigms, theory, or approaches in the literature that other methods are unable to provide

## 3. Results

### 3.1. The Growth Trajectory and Geographic Distribution of Research on International Students’ Mental Health

**Figure 2:**
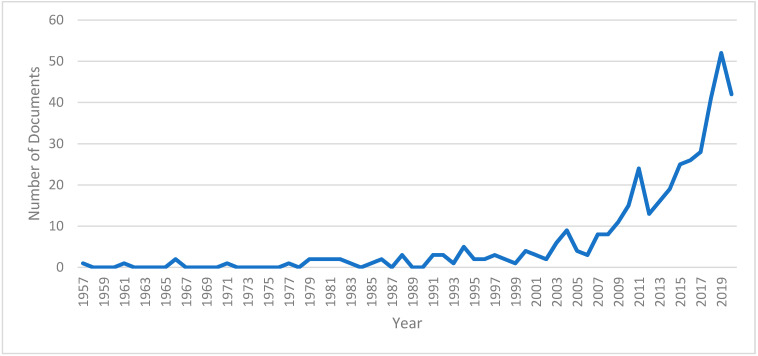
The number of documents on international students’ mental health.

The bibliometric analysis of 402 documents related to international students’ mental health problems has shown that, on average, the annual growth rate of research on international students’ mental health was 9.31%. The 402 documents were allocated across 46 countries, including countries from various regions of the world (e.g., the USA, the UK, Australia, China, South Africa). However, the majority of publications on this topic were contributed by developed Anglo-Saxon countries such as the United States (*n* = 147, 36.57%), Australia (*n* = 65, 16.17%), and the United Kingdom (*n* = 46, 11.44%), which are also three of the most developed countries with open policies for international students [20]. Besides these Anglo-Saxon countries, countries in the Asian continent—China (4.73%), Malaysia (4.98%), Japan (3.98%), and South Korea (3.23%)—were also high on the countries ranking, contributing a total of 68 documents (16.92%) from 1957 to 2019. Not only do these Asian countries export a lot of international students, but recent research has also shown that Asia has become one of the world’s new educational hubs, attracting more and more international students [21,22,23,24]. By looking at the number of publications from 1957 to 2019 (Figure 2), it can be seen that after 43 years (from 1957 to 2000), the number of research on MHIS was very limited, amounting to only 11.69% of the total number of publications (*n* = 47). However, after the turn of the 20th century, it seems like the importance of international students’ mental health was being properly recognized, which resulted in a dramatic increase in the number of publications (*n* = 355, representing 88.31% of the total) in only 19 years.

### 3.2. Identifying the Major Topic Clusters and Highly Influential Papers That Contributed to the Literature on MHIS

In order to answer the second research question, we combined two assessment methods to provide a quantitative and qualitative overview of the literature. First, we ranked the most cited papers within the literature and across scientific fields to provide the readers with bibliographic information on the ten most influential papers. Next, a co-citation analysis was used to draw a clustered network of science mapping the publications in the literature, which identified six major clusters of research topics and groups of authors working on the respective topics. According to Zupic & Čater [25], these clusters can also be interpreted as the schools of thought within the literature. Subsequent research on international students’ mental health within the areas covered by these clusters can use this map to look up the influential research within the relevant topic.

The co-citation analysis provided a science mapping of 255 documents with at least one citation, which created six main clusters within the literature. Across 255 documents, there were a total of 6493 citations, yielding an average of 25 citations per document. When taking into account the papers without any citations, the average citation number per paper was 16. Column 4 of Table 1 shows the global citation (GC), which is the number of citations in a paper. Meanwhile, column 3 shows the local citation (LC) of each paper, which is the number of citations in a paper in a reference list to other papers within the collection (see [26]). While GC can be interpreted as the overall influence of the document across scientific disciplines, LC is the influence of a paper on the examined pool of documents, showing the overall influence of the paper on the literature about international students’ mental health. Due to the stronger focus among academics on local citations.

Table 1 was sorted based on the local citation ranking, and a subsequent in-depth analysis of this measure was focused.

**Figure 3:**
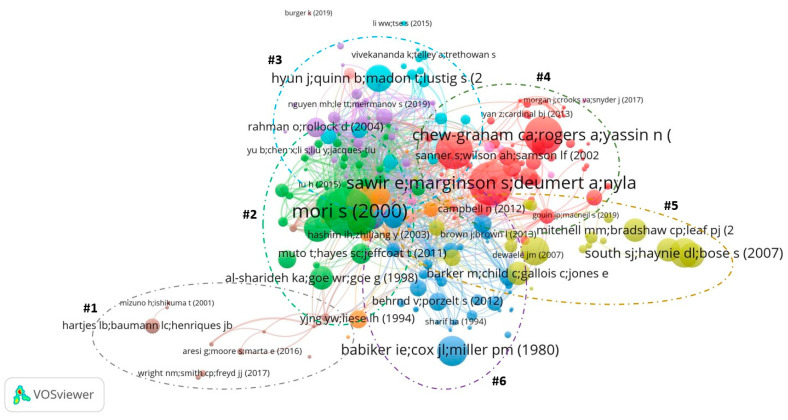
Science mapping of 255 documents based on co-citation analysis. The names of the clusters, from one to six, are (1) Community, (2) Mental Health Problems, (3) Mental Health Service, (4) Linguistic Problem, (5) Mental Acculturation, and (6) Social Acculturation.

The goal of co-citation analysis is to unveil the major topics behind the literature on international mental health by revealing the papers that co-cited each other [13]. Due to the lack of papers in the dataset, we did not impose any threshold for the number of co-citations and displayed every paper that has at least one citation. Among the total of 402 documents examined, 255 documents with at least one citation are shown in Figure 3. While the size of the node represented the number of citations, the number of links between nodes illustrated the co-citation pattern of papers within the literature.

Another feature of the co-citation method are the clusters formed by papers that share the same links. These clusters revealed the so-called schools of thought within the literature, which provided a general overview of the major topics [10]. As Figure 3 has shown, most of the papers in the literature were within more than one topic cluster. Therefore, by examining the most influential and unique documents of each cluster, we can have a glimpse of the major topics within the literature.

The first cluster represents a school of thought named *Community,* which focused on research topics related to the mental/behavioral issues of international students within their community. Researchers in this field often examined a certain group of international students, such as Asian students in Japan [35], Italian students [36], and American students [37,38]. Furthermore, within this school of thought, risky behaviors (e.g., alcoholic problems) as well as helpful behaviors creativity in Ballentine [39], that happened within the international students’ community were also being investigated. The school of thought also explored the vulnerabilities of the international student community when interacting with other stakeholders. For example, a negative external factor such as the lack of support from the educational institution [40], and a higher risk of becoming the victim of sexual harassment/assault and coercion [41], were frequently faced by a certain community of international students.

The second cluster focused more directly on the *Mental Health Problems* of international students. The research on this school of thought acknowledged that international students are a vulnerable group with distinct mental health concerns [6]. A large portion of the research sought to identify risk factors on students’ physical and mental health and their well-being [42,43].

The third cluster was the school of thought termed *Mental Health Service.* The research on these topics aligned with another school of thought in medical research called Health Service research [44]. The research on this school of thought focused on identifying vulnerable groups [45,46,47], risk factors [48,49], awareness and need of service [28], actual service use, and treatment effectiveness [50,51]. The research on this cluster mostly concluded that international students under-utilized the mental health service due to various reasons, such as cultural taboos and acculturation. Therefore, it is critical for educational institutions and policy-makers to develop suitable preventive measures and treatments that suit the needs and problems of international students.

The fourth cluster was directed toward a specific problem that is unique to international students compared to domestic students, which is the *Linguistic Problem*. Although most international study destinations require a certain level of language proficiency for admission eligibility, a large portion of international students still face linguistic problems when adapting to the study destination. The research on this topic pointed to the environmental negative consequences of linguistic obstacles, such as problems in adapting to the social and cultural characteristics [52,53] of the workplace/study [54] and even the legal environment [55] of the study destinations. Furthermore, struggling with the language and, subsequently, the cultural adaptation also led to internal problems such as stress and anxiety [54,56]. It is also noticeable that the research on this topic was adopted from the approach of the educational institution and policy-makers [55,57,58], indicating that these stakeholders were focusing more on examining and solving this problem.

Similar to the previous one, the fifth school of thought focused on an adaptive problem that concerns mostly international students, which was *Mental Acculturation*. How well one adjusts to the study destination’s culture can be a crucial factor related to one’s mental health and overall well-being during the study period. Failing to adapt to the environment led to stress and anxiety in international students [59], while good adaptive behaviors can be very beneficial to their academic performance and well-being [60]. Here, culture adjustment referred not only to adaptive changes to the lifestyle but also to the academic environment [61]. The research on this topic also examined the differences in the coping behavior of international students when studying abroad between cultural aspects such as individualism and collectivism (see [62]), and religions [63]. Specifying these differences might help education institutions and researchers better identify the needs of each international students group.

The final cluster referred to the school of thought called *Social Acculturation*, which was closely related to the fifth cluster. While the previous cluster mostly referred to the mental impacts from success or failure in acculturation, this school of thought examined the social aspects of the adjustment process. The research on social acculturation focused especially on the reverse culture shock of international student returnees, which is how well they adapt to the social life in the origin country after finishing their study abroad [64,65]. Other social acculturation problems were also examined, such as how maladaptations to the workload and finance management in a new environment [37,66,67] can cause stress and anxiety in students. The research on this topic showed that one of the most problematic adjustments to the new culture is food [68,69], which interestingly related to one sense of culture, community, and social life. The inability to adapt to the food in the study destination might be an identifier of acculturation. On the other hand, eating food from the host country has been shown to improve emotional health, well-being, and social life within and outside one’s community [68].

Furthermore, by comparing the number of links within the top-cited paper in the science mapping, we can identify the papers that have a wide large impact and connections with other schools of thought within the literature. The authors/papers with these two characteristics, often placed at the center of the co-citation map, are considered as boundary-spanning units, which play a crucial role in developing the literature by increasing the influence and reducing the scientific proximity between papers among different schools of thought [70,71]. On top of the citations count table, there is the research conducted by Mori [6] with a total link strength of 100. However, the 6th highest paper in terms of citations count, Lee et al. [31], despite having fewer citations (167), has a higher total link strength (132) than the other papers in the top five, illustrating an interesting case of a strong connector with lesser influences.

### 3.3. A Timeline of the Most Researched Keywords in the Literature on MHIS

As the previous research question was mainly based on the intellectual network to provide a map of the foundational research and topics in the literature, this section focused further on the trending topics that are currently being addressed in the literature. The co-occurrence analysis with time identification described the evolution of the literature across time. Therefore, researchers new to the literature are hereby provided with a historical account of previous topics as well as the newest trends on international students’ mental health. Occurrence analysis showed that the most used keywords beside international students (131 co-occurrences) and mental health itself (37 co-occurrences) are acculturation (29 co-occurrences), acculturative stress (25 co-occurrences), and depression (20 co-occurrences).

As can be seen from the color scale of the graph, which represented the year of publication, most of the papers with these prominent keywords were published within the 2014–2016 period. Besides focusing on the symptoms of different mental disorders, the research within this period also examined the general factors related to mental health, such as acculturation [60,72,73,74,75] and well-being [76,77,78], that might provide a more holistic and generalizable explanation [79]. By examining the keywords from 2018 onward, we noticed two major trends in the research on international students. First, there was a stronger emphasis on a group of students or study destination. For example, in recent years, the research on international students’ mental health showed a strong focus on the Chinese [80,81,82,83], Japanese [84,85], and medical international students [86,87,88]. Meanwhile, Australia has received more attention as a study destination [83,89,90,91]. This science mapping showed a strong trend in the identification of the vulnerable group of students and mental health while studying abroad. As international students’ mental health is influenced by the interaction between the students’ mindset and the surrounding environment, this science mapping showed that recent research is focusing more on investigating these specific interactions, that have not been explored before. Secondly, the newer keywords on the science mapping also showed a stronger emphasis on modern issues that students are facing during their study: the focus on changes in the environment and behavioral problems such as problematic social media use [2,92,93], social drinking and gambling [38], [90,94] has increased in recent years. Environmental changes and adaptation problems such as COVID-19 [9,91,95], coping strategies [96,97,98], and homesickness [68,99] are also being emphasized. It can also be noticed that, in contrast to the purple nodes, which clustered around the top of the map, the light green nodes were distributed evenly around the map. Furthermore, yellow nodes appeared across the outskirts of the map, indicating that the literature is expanding toward different directions.

## 4. Discussion and Conclusions

International students in higher education have been identified as vulnerable to mental health issues such as depression, anxiety, loneliness, and stress [6]. Furthermore, due to their distinctive social and cultural diversity, applying previous results about local students’ mental health is undesirable and might lead to poor decisions regarding this important issue [3,5]. Therefore, this paper utilized the bibliometric analysis technique to provide the first review of research on international students’ mental health from 1957 to 2019. The bibliometric approach is highly advantageous because of its ability to visualize the relationship between intellectual units within a scientific topic and has been used across different scientific disciples [10,12,71,100]. From the descriptive analysis of 402 Scopus-indexed documents, it has been shown that there was an explosive growth in the number of publications after 2000, published mostly by authors in Anglo-Saxon developed countries. However, the issue also received a substantial amount of attention from countries in Asia, indicating a growing demand for research topics in this region.

The second research question was addressed by co-citation analysis, which unveiled six major schools of thought within the literature. These major topics focused on issues that are uniquely and closely related to international students’ mental health, such as the community, mental problems, mental health services, linguistic problems, and acculturation. Originally, according to White and McCain [70], and later restated by Det Udomsap and Hallinger [71], the co-citation mapping technique not only intuitively showed the clusters but also addressed the quantity and influence of papers that acted as boundary-spanning units, expanding the literature and connecting the clusters within the literature. In line with previous opinions about the increasingly important role of the literature (e.g., see [101,102]), the analysis also showed that the topic of international students’ mental health has a high average number of citations per paper, and that there is a high interest in the topic across different scientific disciplines.

The last question was addressed by co-occurrence analysis, which showed that the evolution of the research on international students’ mental health is expanding quickly and exploring new research directions. The research focusing on the symptoms of disorders (e.g., [4,103]) and general factors such as acculturation [60,72,73] accounted for the prevailing research keywords in the first half of the 2010s, whereas the second half of the decade witnessed a stronger focus on contemporary problems such as COVID-19 [8,91,95], the Internet and social media use [2,104]. Such research topics, relating to modern issues and recent changes in society, are thus gaining more traction and should be given more attention.

This study makes several contributions, both in terms of theory and practice. First, the finding that MHIS has received increasing attention from academic scholars from different countries suggests that this issue should be further elaborated. As international students have become an indispensable component of the higher education landscape, and as they have distinct concerns regarding mental health, studies on MHIS should be considered as a distinct track, which differs from the track of the studies on domestic students’ mental health [105].

**Figure 4:**
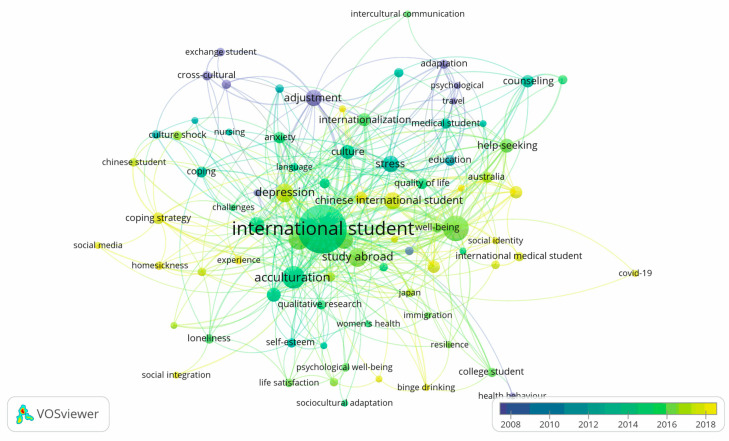
A network of co-occurrence analysis of keywords with at least three occurrences between 1957 and 2019. The color of each keyword node represents the average publication year of papers containing that keyword.

Second, our identification of major topic clusters, highly influential papers, and the most researched keywords on MHIS are paramount for future scholars when they consider conducting research on MHIS. For example, a future scholar on MHIS may select either one of six research clusters shown in Figure 4 (these are: Community, Mental Health Problems, Mental Health Service, Linguistic Problem, Mental Acculturation, Social Acculturation) as their research topic. Furthermore, the most researched keywords, as shown in the Figure 4, especially those in yellow color, should without doubt be taken into consideration by MHIS scholars in their upcoming research.

## 5. Limitations

As limitations are unavoidable in any research [106], this study is not an exceptional case. First, the bibliometric analysis methods used in this research focus more on the quantitative and descriptive overview of the literature than on providing a qualitative summary of research findings. Therefore, they only serve as preliminary guidance for new research on the topic. Thus, unlike traditional meta-analyses, we cannot address problems about the literature such as effect sizes and publication bias [106]. It is highly recommended that, after the readers have identified the relevant information from this paper, an investigation of the literature main findings be conducted.

The second criticism is related to the use of only Scopus-indexed documents. While Scopus provides a wider coverage of research articles, the Web of Science has a huge collection of book chapters and conference papers that might substantially change the science mapping of research [16]. Although it is debatable whether one database is better than another, the use of the Web of Science database was more predominant in similar educational science mapping research. It is preferable for future research to utilize multiple databases to have a more comprehensive dataset of the literature (e.g., see [36,107,108,109]). Furthermore, one can also probe for more data by collecting in-press research and accepted manuscripts from the researchers within the field. Due to logistic issues, we only have access to the Scopus dataset, which greatly hindered the number of documents in the dataset and can potentially obstruct further observations about the research topic.

## Figures and Tables

**Table 1:** Top 10 most locally cited papers in the literature. The number in parenthesis is the respective ranking of the paper with regard to the criteria in the column.

No	Authors	Local Citation (Ranking)	Global Citation (GC)	LC/GC Ratio
1	Mori [6]	53 (1)	370 (1)	0.143
2	Sandhu & Asrabadi [27]	31 (2)	265 (3)	0.117
3	Hyun et al. [28]	23 (3)	114 (13)	0.202
4	Sawir et al. [29]	18 (4)	294 (2)	0.061
5	Han et al. [4]	18 (5)	41 (41)	0.439
6	Zhang & Dixon [30]	16 (6)	117 (12)	0.137
7	Lee et al. [31]	12 (7)	167 (6)	0.072
8	Wang et al. [32]	11 (8)	71 (21)	0.155
9	Sherry et al. [33]	10 (9)	211 (5)	0.047
10	Misra & Castillo [34]	9 (10)	130 (10)	0.069

## Data Availability

The data presented in this study are available on request from the corresponding author.
